# From Founder Events to Genetic Recovery? Three Decades of Wolf Expansion Across the Italian Alps

**DOI:** 10.1111/mec.70639

**Published:** 2026-11-14

**Authors:** Francesca Rolle, Kristine L. Pilgrim, Elisa Avanzinelli, Michael K. Schwartz, Francesca Marucco

**Affiliations:** ^1^ Department of Life Sciences and Systems Biology University of Torino Torino Italy; ^2^ USDA National Genomics Center for Wildlife and Fish Conservation Missoula Montana USA; ^3^ Large Carnivore Center Ente di Gestione delle Aree Protette delle Alpi Marittime Valdieri Italy

**Keywords:** connectivity, effective population size (N_e_), founder events, genetic monitoring, population structure, wolf ( *Canis lupus* )

## Abstract

Understanding how genetic diversity and population structure evolve during natural recolonization is critical for the conservation of wide‐ranging carnivores in human‐dominated landscapes. We analysed 27 years of continuous genetic monitoring (1998–2024) of the naturally expanding Italian Alpine wolf ( *Canis lupus* ) population, providing one of the most comprehensive temporal datasets for any recovering large carnivore in Europe. Using > 6500 samples and 1584 genetically identified individuals, we evaluated population responses through time to two different founder events and assessed long‐term trends in genetic diversity and population structure, considering the effects of connectivity on the estimated effective population size (N̂_e_) over time. We found persistent genetic distinctiveness between the western and eastern Italian Alpine regions, despite ongoing demographic expansion. The western Alps currently act as a primary source of genetic diversity, with high allelic richness, within‐region migration rates and a detectable increase in N̂_e_ over the last 10 years. The eastern Alps still exhibit low genetic diversity, mirroring the trajectory observed in the western Alps during the early stages of recolonization and signatures of recent founder events. Contemporary gene flow across the Italian Alps remains asymmetric and extremely limited, potentially constraining long‐term viability. Pack number proved to be a reliable proxy for effective population size in the Italian Alpine wolves, particularly during the early phase of population expansion and when spatial population structure was accounted for by analysing the eastern and western subpopulations separately. Our results demonstrate that long‐term demographic recovery alone does not necessarily translate into genetic recovery, underlining the need for continued genetic monitoring to inform conservation strategies that maintain connectivity and adaptive potential in recolonizing large carnivores.

## 1 Introduction

Understanding the genetic patterns associated with population decline and expansion has become increasingly important for conservation biology, particularly as species respond to environmental changes and management interventions (Theissinger et al. 2023; Hohenlohe et al. 2021; Willi et al. 2022). Recent global assessments indicate that within‐population genetic diversity is being lost over short timescales (Shaw et al. 2025), making temporal genetic studies increasingly urgent for understanding and offsetting population declines and recoveries. Genetic data offer insights into demographic and evolutionary processes that are difficult or impossible to obtain through traditional methods alone (Schwartz et al. 2007; Hoban et al. 2021). By analysing genetic markers across populations and through time, it becomes possible to estimate key indicators of population viability and adaptive potential, such as effective population size, levels of inbreeding, genetic diversity, population structure and gene flow (Lowe and Allendorf 2010; Luikart et al. 2003), widely recognized as essential for species' conservation and management (Hohenlohe et al. 2021). Indeed, genetic monitoring, or the quantification of temporal changes in population genetic metrics (Schwartz et al. 2007; Pazhenkova et al. 2025), provides valuable tools for conservation. Beyond detecting warning signals of population decline, loss of connectivity, or genetic erosion in threatened populations (Luikart et al. 2021; Leroy et al. 2018), genetic monitoring can also reveal the consequences of population growth, following colonization, reintroduction or range expansion (Tallmon et al. 2012; Jensen et al. 2018; De Barba et al. 2010).

The genetic consequences of these population dynamics are particularly pronounced in small, isolated, or recovering populations, where stochastic processes, such as genetic drift and inbreeding, can rapidly erode genetic diversity (Reed and Frankham 2003; Fontsere et al. 2025). Human pressure further amplifies these effects by leading to population isolation, as urban development and habitat fragmentation restrict natural gene flow. Following founder events, genetic diversity can be drastically reduced compared to source populations (Allendorf et al. 2022; Fraik et al. 2025). Serial founder events during colonization processes can create spatial patterns of genetic diversity, although over time, if populations grow and connectivity is restored, these initial genetic signatures may resolve, leading to recovery of diversity across landscapes (Keller et al. 2012). Documenting spatio‐temporal genetic dynamics is therefore essential to understand whether recovering populations are on trajectories toward long‐term viability or remain vulnerable to genetic stochasticity, especially in the highly fragmented landscapes of Europe.

Wolves ( *Canis lupus* ) offer interesting model systems to study these population genetic processes. As wolves are currently recolonizing their historic ranges across human‐dominated landscapes in Europe (Di Bernardi et al. 2025; Chapron et al. 2014) and North America (Hendricks et al. 2019), they represent a species of crucial management interest due to important ecological, cultural and political implications. Their well‐defined social structure, characterized by pack formation from monogamous breeding pairs, territoriality and dispersal, offers unique opportunities to understand how behavioural ecology and social organization shape genetic diversity and population structure over time. In the reintroduced Yellowstone National Park wolf population, one of the most intensively studied systems, long‐term genetic data have revealed that dispersal, inbreeding avoidance mechanisms, and social structure interact to maintain genetic diversity (vonholdt et al. 2008). In contrast, wolf recolonization in anthropized Europe occurs in highly fragmented habitats shaped by centuries of intensive land use (Kuijper et al. 2024). Vilà et al. (2003) showed how, in the geographically isolated Scandinavian wolf population, migration events, even at a very low rate, led to the rescue and recovery of the small and inbred population. In Germany, the naturally expanding Central European wolf population showed dispersal dynamics and connectivity to be crucial for rapid recovery, despite past population bottlenecks (Jarausch et al. 2021). Similarly, the Italian peninsular wolf population has served as another model to document the effects of a natural expansion, where long‐distance dispersal by individuals and the establishment of stable packs enabled the northward expansion toward the Alps following bottleneck events (Caniglia et al. 2014; Fabbri et al. 2007). Wolves naturally recolonized the western Alps expanding from the Apennine population at the end of the 90s, following their extirpation in the early 20th century (Marucco et al. 2009; Fabbri et al. 2007), with evidence of a first founder effect, a moderate bottleneck, and unidirectional gene flow (Fabbri et al. 2007). Following the population expansion in the Western side of the Alps, a stochastic colonization event of only two individuals in the Eastern Alps occurred in 2012, where wolves were previously absent (Ražen et al. 2016), accelerating the Alpine recolonization of the central‐eastern Alps. Although demographic recovery has been extensively documented (Marucco et al. 2009; Marucco, Pilgrim, et al. 2022; Marucco, Reinhardt, et al. 2023), genetic assessments have been limited to the early stages of Alpine recolonization (Fabbri et al. 2007; Lucchini et al. 2002) and it remains unclear how genetic diversity, population structure and connectivity have evolved over the past three decades of this natural expansion to lead to a long‐term genetic recovery.

We conducted 27 years of continuous, systematic genetic monitoring of the natural expansion of the Italian Alpine wolf population, supported by long‐term regional and international funding programs. The Italian Alpine wolf population has experienced no direct management interventions, providing a rare opportunity to explore how stochastic processes, including founder effects and inbreeding, naturally evolve during long‐term recolonization across the highly fragmented Alpine landscape. We address four major questions: (1) How has the genetic structure of the Italian wolf population evolved over 3 decades of natural expansion in the Alps, and has the pack‐based social organization left detectable effects on the overall population's genetic structure through time? (2) How have the two distinct founder effects, derived from temporally separate wolf colonization events in Italy, attenuated over time, and what consequences have these processes had for the wolf population across the anthropized Alpine landscape? (3) Has genetic connectivity between the western and eastern Alpine wolf populations changed over time following the most recent stochastic colonization event? (4) How the estimated effective population size (N̂_e_) trend evolved compared to the estimated pack number in wolves, and how can this inform evidence‐based conservation management in the Alps?

## 2 Materials and Methods

### 2.1 Sampling Design

This study covers the entire Italian Alps, spanning approximately 51,500 km^2^ (Figure 1). Between 1998 and 2024, a continuous genetic monitoring of the naturally expanding Italian Alpine wolf population was conducted through coordinated regional and international long‐term projects funded by Regional Administrations and by two European LIFE programs. All sampling followed standardized non‐invasive protocols (Marucco, Boiani, et al. 2023) implemented by a network of trained field operators. Systematic non‐invasive genetic sampling along transects, which mainly focused on scats collection, was conducted annually following the species' reproductive cycle (May 1, Year^X^‐April 30, Year^X + 1^), with intensified effort during winter months (November–April) when snow tracking facilitated sample detection and collection (Marucco et al. 2011). Sampling effort was stratified within 10 × 10 km grid cells covering the study area, according to the probability of wolf presence, based on prior indications suggesting the expected occurrence of the species, with higher survey intensity in confirmed wolf presence areas. Specifically, winter monitoring in these cells involved monthly surveys (six repetitions) along standardized transects to ensure robust detection (Marucco, Boiani, et al. 2023). In addition to systematic sampling, opportunistic sampling was carried out everywhere in the study area, including cells where wolf presence was not documented. Collected materials occasionally included hair, blood, urine and tissue from dead individuals, in addition to scats. As the wolf population expanded, systematic transects were established in newly colonized cells (details in Marucco, Boiani, et al. 2023). This adaptive design ensured continuous spatial coverage of the study area with systematic sampling from 1998 to 2010, and in the monitoring periods 2014–2015, 2015–2016, 2017–2018, 2020–2021 and 2023–2024, consistent with the national wolf monitoring protocol adopted in Italy (Marucco et al. 2020). In all other years, monitoring was conducted opportunistically. The pedigree reconstruction and temporal evolution of the wolf packs in the study area is reported in Marucco, Avanzinelli, et al. (2022). It is worth noting that the genetic samples from the eastern Alps used in this study cover the majority of the wolf population distribution in the area, but do not include the northern region of Trentino Alto Adige. Although monitoring activities are conducted within a broader collaborative framework, samples from this region were analysed by a different laboratory using slightly different laboratory procedures and genotyping platforms. Consequently, the resulting genotypes could not be directly integrated into the present dataset without specific cross‐laboratory calibration.

**FIGURE 1:**
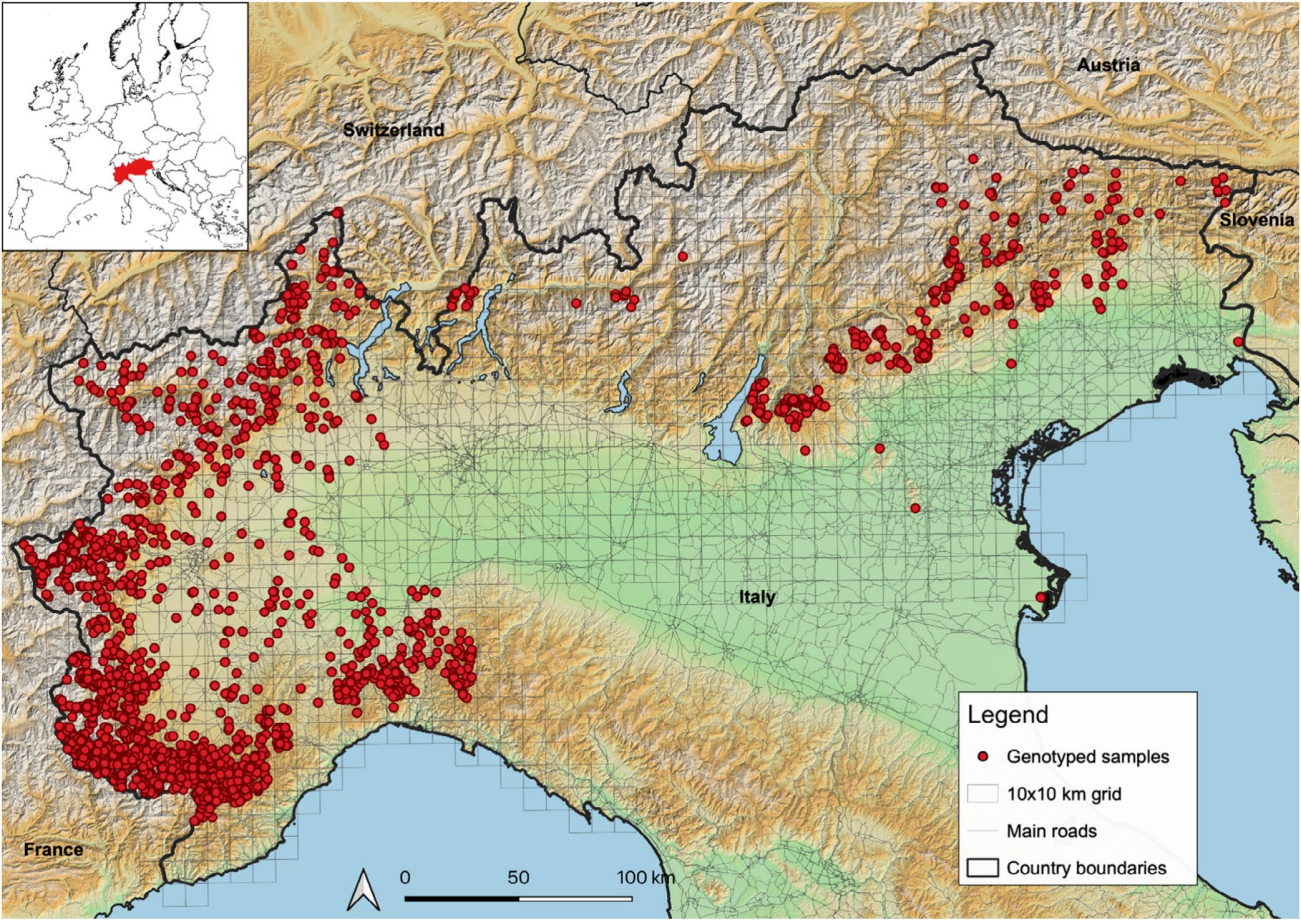
Spatial distribution of successfully genotyped samples non‐invasively collected along standardized transects within a 10 × 10 km grid and through opportunistic sampling across the Italian Alps between 1998 and 2024.

### 2.2 DNA Extraction and Microsatellite Analysis

We extracted DNA from scats using the QIAamp DNA Stool Minikit (QIAGEN Inc., Germany), and from hair, urine, blood and tissue samples from dead recovered wolves using the DNeasy Blood and Tissue Kits (Qiagen Inc.), following previously described standard protocols in Marucco et al. (2009) and Caniglia et al. (2021). DNA samples were subjected to species identification and individual genotyping. Mitochondrial DNA was used to differentiate wolf samples from those of other carnivores and to assign haplotypes, following Marucco et al. (2009). Wolf samples were genotyped and sexed using laboratory‐specific panels of 10 (samples from 1998 to 2010) or 16 (2013 to 2024) microsatellite loci, previously validated in canid genetic studies (dinucleotide repeats: CPH2, CPH4, CPH5, CPH8, CPH12 and tetranucleotide repeats: FH2004, FH2079, FH2088, FH2096, FH2137, FH2054, FH2140, FH2161, PEZ17, U250, U253), following the ISPRA National Genetics Laboratory protocol (Caniglia et al. 2014, 2021). Samples were genotyped at the USFS National Genomics Center. Microsatellite data were checked for quality and for potential allelic dropout or false alleles. Individuals with successful amplification at fewer than eight loci were excluded from further analyses due to insufficient genotypic resolution. Individual multilocus genotypes were compared using Dropout v2.3.3 (McKelvey and Schwartz 2005) to identify and remove duplicate profiles and to estimate the probability of identity (PID and PIDsib). This follows the error checking protocols used with other non‐invasive genetic sampling monitoring efforts (e.g., Schwartz et al. 2006; Fraik et al. 2025; Day et al. 2024). Loci were checked for null alleles using program Cervus 3.0 (https://cervus.software.informer.com/3.0). An additional error‐checking phase was implemented based on pedigree reconstruction by local area across all years of the monitoring period, during which genetic data was also compared to other independent field information, such as documented presence of packs by camera trapping, snow‐tracking, wolf‐howling, GPS‐collared wolves and dead recoveries. When inconsistencies with the expected pedigree suggested a potential error, the corresponding samples were re‐amplified and, when necessary, re‐genotyped to confirm or correct the genotype.

### 2.3 Population Genetics Analyses

Genotypic data quality was assessed prior to downstream analyses. We tested for departures from Hardy–Weinberg equilibrium (HWE) using Genepop v. 4.5.1 (Rousset 2008), with dememorization number = 5000; number of batches = 1000; number of iterations per batch = 5000. Standard measures of genetic diversity, including the number of alleles per locus (Na), observed (Ho) and expected (He) heterozygosity, allelic richness (Ar) and the polymorphism information content (PIC), were calculated as implemented in the hierfstat package (Goudet 2005) on R (R Core Team 2025).

To meet the first objective, estimating the genetic structure of Italian Alpine wolves and assessing whether pack‐based social organization left detectable signatures over time, we employed the Bayesian clustering algorithm implemented in STRUCTURE v2.3.4 (Pritchard et al. 2000). This method uses a Markov chain Monte Carlo (MCMC) procedure to estimate the number of clusters (*K*), without prior information on sampling location. Analyses were performed under an admixture model with correlated allele frequencies. For each candidate value of *K* (2–10), five independent runs were conducted. Each run consisted of a burn‐in period of 100,000 iterations, followed by 1,000,000 MCMC iterations. Convergence was assessed by visual inspection of likelihood plots and by comparing posterior probabilities among runs. The most likely *K* was determined using the Evanno Δ*K* method (Evanno et al. 2005), implemented in Structure Harvester (Earl and vonHoldt 2012). Population structure analyses were performed on the full dataset (including all individuals and a subset of reproductive individuals to explore the influence of pack structure) and separately for each year. The subset of reproductive individuals was identified based on the pedigree reconstruction reported in Marucco, Reinhardt, et al. (2023); Marucco, Boiani, et al. (2023). Specifically, breeding individuals were identified as members of reproductive units, which were defined by either the documented occurrence of pups or the presence of at least three wolves travelling together and holding a territory.

To complete the second objective, detecting consequences of the founder effects on the wolf Alpine population (late 1990s in the west and ~2014 in the east), we first derived a continuous spatial surface of allelic richness by applying inverse distance weighting interpolation in the R package phylin (Tarroso et al. 2015). We then calculated FIS, as a measure of departure from expected heterozygosity, using the hierfstat package (Goudet 2005). Samples were grouped into consecutive 3‐year periods and analyses were conducted separately for the western and eastern Alps. We identified private alleles by analysing genotypic data separately for the western and eastern portions of the Italian Alpine population, following the previous genetic structure analyses. Private alleles were defined as those occurring in only one area (i.e., western or eastern Alps) and completely absent in the other. We applied Slatkin's private allele method (Barton and Slatkin 1986) for estimating gene flow as implemented in Genepop 4.8.2. Additionally, contemporary levels of gene flow between the west and the east of the Italian Alps were assessed using BayesAss v3.0.5 (Wilson and Rannala 2003), which estimates the fraction of immigrants in a population using Bayesian inference. We performed 10 runs using 10,000,000 iterations and a burn‐in of 1,000,000. The mixing parameters DeltaA, DeltaF and DeltaM were set to 0.05, 0.05 and 0.10 respectively, to achieve recommended acceptance rates (Wilson and Rannala 2003). Each run was initialized with different starting‐seed values to achieve consistency of mean parameter estimates between runs (Wilson and Rannala 2003). To increase the statistical power and inference reliability of BayesAss output, we followed Meirmans (2014) further suggestions and did not average results, instead reporting the most biologically meaningful and repeated results. Convergence of the chains was validated using Tracer v1.6 (Rambaut et al. 2014). We further studied gene flow in the Alps by applying the estimated effective migration surfaces model (EEMS; Petkova et al. 2016), which is built to visualize patterns of genetic differentiation over shorter distances by fitting a continuous surface of estimated gene flow across the landscape. In addition to effective migration rates, EEMS estimates effective diversity rates, which describe the expected genetic differentiation between two distinct individuals sampled from the same deme (i.e., the within‐deme component of genetic variation; Petkova et al. 2016). Areas with higher effective diversity indicate greater within‐location genetic differentiation than the global average, whereas lower values indicate reduced within‐location genetic differentiation. We set the EEMS parameters such that the acceptance proportions were from 20% to 30%, as recommended in Petkova et al. (2016). Each EEMS run consisted of 2,000,000 MCMC iterations, with the first 1,000,000 discarded as burn‐in, and sampled every 9999 iterations. We applied the EEMS model to the complete dataset using 10 microsatellite loci and to the samples collected between 2014 and 2024 using 16 microsatellite loci.

To estimate the effective population size (N̂_e_), we first divided the Italian wolf population spatially, following previously identified genetic clusters as determined by the population structure analysis, and temporally into generations. Generation length in European wolves varies across studies depending on the population and the metric used (e.g., average reproductive age, Aspi et al. 2006; or mean age of female breeders, Battilani et al. 2025). Published estimates range from 3 years (e.g., Sastre et al. 2011 in the Iberian Peninsula; Aspi et al. 2006 in Finland; Lucchini et al. 2004 in the Italian Apennines) to 4 years (Pérez‐Sorribes et al. 2024 in Scandinavia; Battilani et al. 2025 in the Italian peninsula) and up to 5 years (Mergeay et al. 2024 in Germany). To account for this variability and ensure robust Ne estimates, we first used the data spanning 1998–2010 (years with annual systematic sampling) and calculated N̂_e_ using both 3‐ and 4‐year generation lengths (details in Supplementary S1). We also used a third temporal grouping by shifting the starting year by one year to avoid a bias in the interval choice. Following this sensitivity testing we then calculated N̂_e_ for the remaining years, noting that each year group may include individuals of different ages. For each temporal pool we applied four microsatellite‐based Ne calculation methods: the linkage disequilibrium (LD) method of Hill (1981) and Waples (2006), assuming (1) random mating and (2) monogamy, as implemented in NeEstimator V2.1 (Do et al. 2014), and the sibship frequency (SF) method of Jones and Wang (2010) as implemented in COLONY 2.0 under both (3) random mating and (4) non‐random mating assumptions, which reconstructs pack relationships and estimates Ne from the proportion of full and half‐sibling pairs. We compared the Ne estimates to the number of packs, as the reproductive unit identified by either pup occurrences or by at least three individuals travelling together and holding a territory, derived from previously published reports, and available up to 2020–2021 (Wolf Alpine Group 2024; Marucco et al. 2018).

## 3 Results

### 3.1 Descriptive Results

From 1998 to 2024, 22,158 samples were collected across the entire Italian Alps and 6634 of these samples were analysed. Overall, mitochondrial DNA was successfully retrieved from 5632 samples, the vast majority from scats (*n* = 5262), the rest from hair (*n* = 24), blood (*n* = 45), urine (*n* = 15) and tissue (*n* = 387) samples. Samples assigned to dogs (*n* = 202), golden jackals (*n* = 4) and foxes (*n* = 28), based on mtDNA, were excluded from subsequent analyses. 4052 samples were successfully genotyped, allowing for individual identification, the majority of which derived from the western Alps (*n* = 3557), the rest from the eastern Alps (*n* = 495) (Figure 1). Overall, 1694 individuals were identified. Wolf individuals were detected between 1 and 64 times, with a mean of 2.5 detections per individual. Testing our datasets across all available loci showed that < 10% of the loci deviated significantly from HWE (*p* < 0.05), all loci had negligible null allele frequencies of < 0.06 and the estimated per‐genotype error rate was estimated at 1.62%. We calculated standard measures of genetic variation for all 1694 identified individuals, based on the 10 common loci throughout the study period. The mean number of alleles was 10.2, observed heterozygosity was 0.67 and expected heterozygosity was 0.69. The set of loci analysed showed high polymorphic information content (PIC = 0.64). The probability of identity (PID) was 2.9 × 10^–09^ and the probability of identity between siblings (PIDsib) was 2.79 × 10^–04^, indicating that identical genotypes occurring by chance in the population were extremely unlikely.

### 3.2 Genetic Structure of the Italian Alpine Wolf Population

Overall, microsatellite analyses of the Italian Alpine wolf population structure revealed the presence of multiple genetic clusters. Among the tested *K* values (2–10), Δ*K* was highest at *K* = 3 (Δ*K* = 128.05), indicating that the population is most likely structured into three genetic clusters (Figure 2). These results were also supported when STRUCTURE was run on the subset of reproductive individuals only. Two clusters were found in the western Alps, broadly overlapping across a wide geographic area and accounting for 92.7% of individuals sampled in this region, while a third cluster was located in the eastern Alps, accounting for 84.5% of individuals sampled in this region. A total of 21 individuals in the eastern portion of the Alps showed inferred ancestry to the west > 0.60, likely reflecting genetic connectivity with the Western population. Analyses of population structure for the early recolonization period (2004–2010) revealed clusters corresponding to differentiated genetic lineages. Instead, results from the more recent colonization years (2014–2023) primarily highlighted differentiation between western Alps and eastern Alps clusters, without revealing smaller territorial substructure.

**FIGURE 2:**
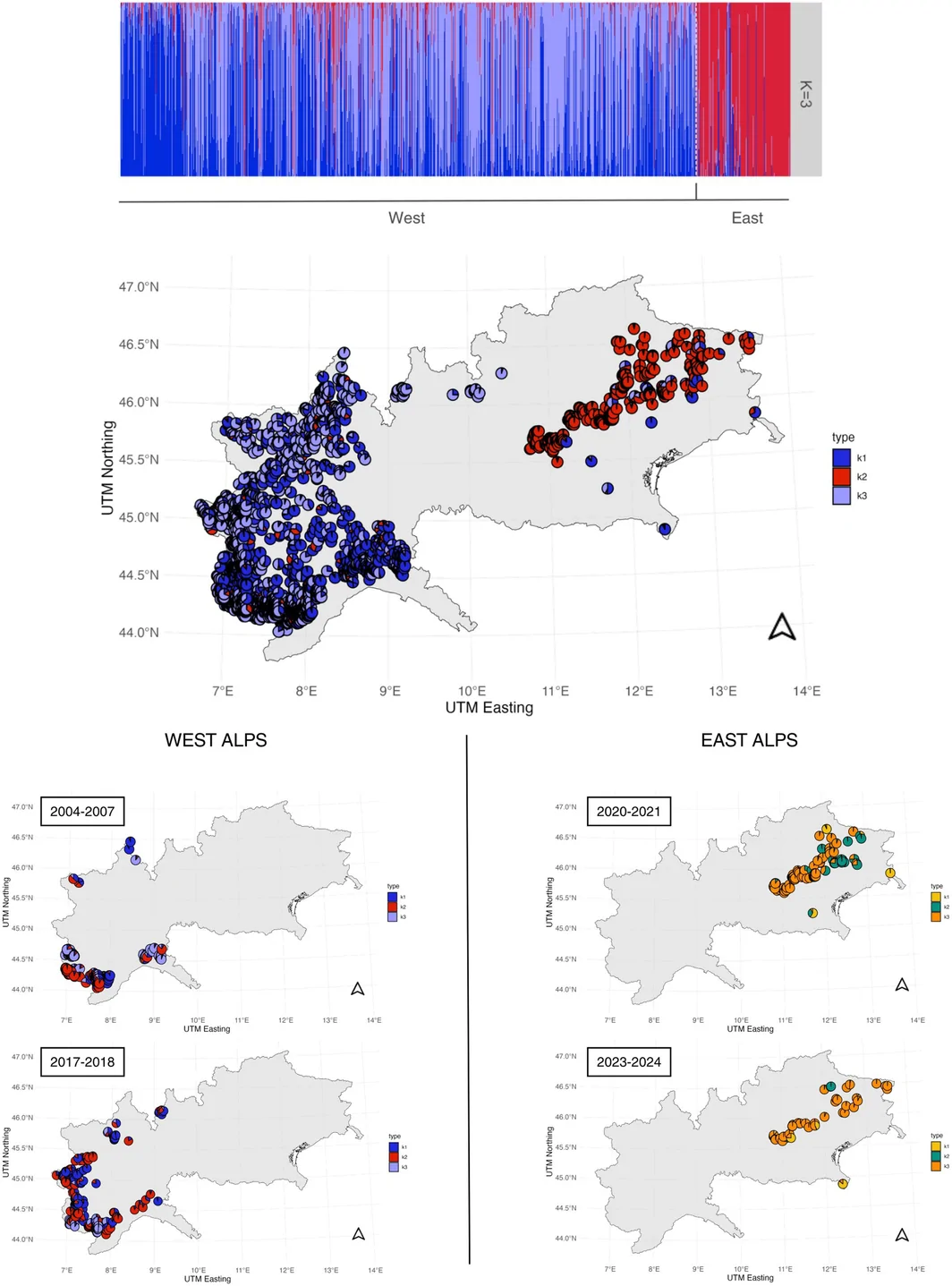
Overall population structure of the Italian Alpine wolves (above) and spatial separation in western (left) and eastern Alps (right) showing decreasing differentiation through meaningful monitoring years. Admixture proportions for each individual are shown as bar plots and spatially explicit pie charts.

### 3.3 Genetic Consequences of Founder Effects in the Alps

Spatiotemporal interpolation of allelic richness (Ar) revealed an overall increase of genetic diversity across the Italian Alps over the 27‐year sampling period (Figure 3). During the early recolonization phases (1998–2009), the wolf population showed low Ar values, ranging from 3.50 ± 0.22 in 1998–1999 to 4.96 ± 0.43 in 2009–2010. Starting from 2014, localized zones of lower Ar are present in the eastern Alps, consistent with the more recent founder event, with Ar = 3.75 ± 0.33 in 2014–15. However, by the most recent sampling period in 2023–2024, Ar increased across most of the distribution, producing a band of comparatively high allelic richness, with higher values reported in the west (Ar = 5.91 ± 0.79) and increasing in the east (Ar = 4.82 ± 0.46).

**FIGURE 3:**
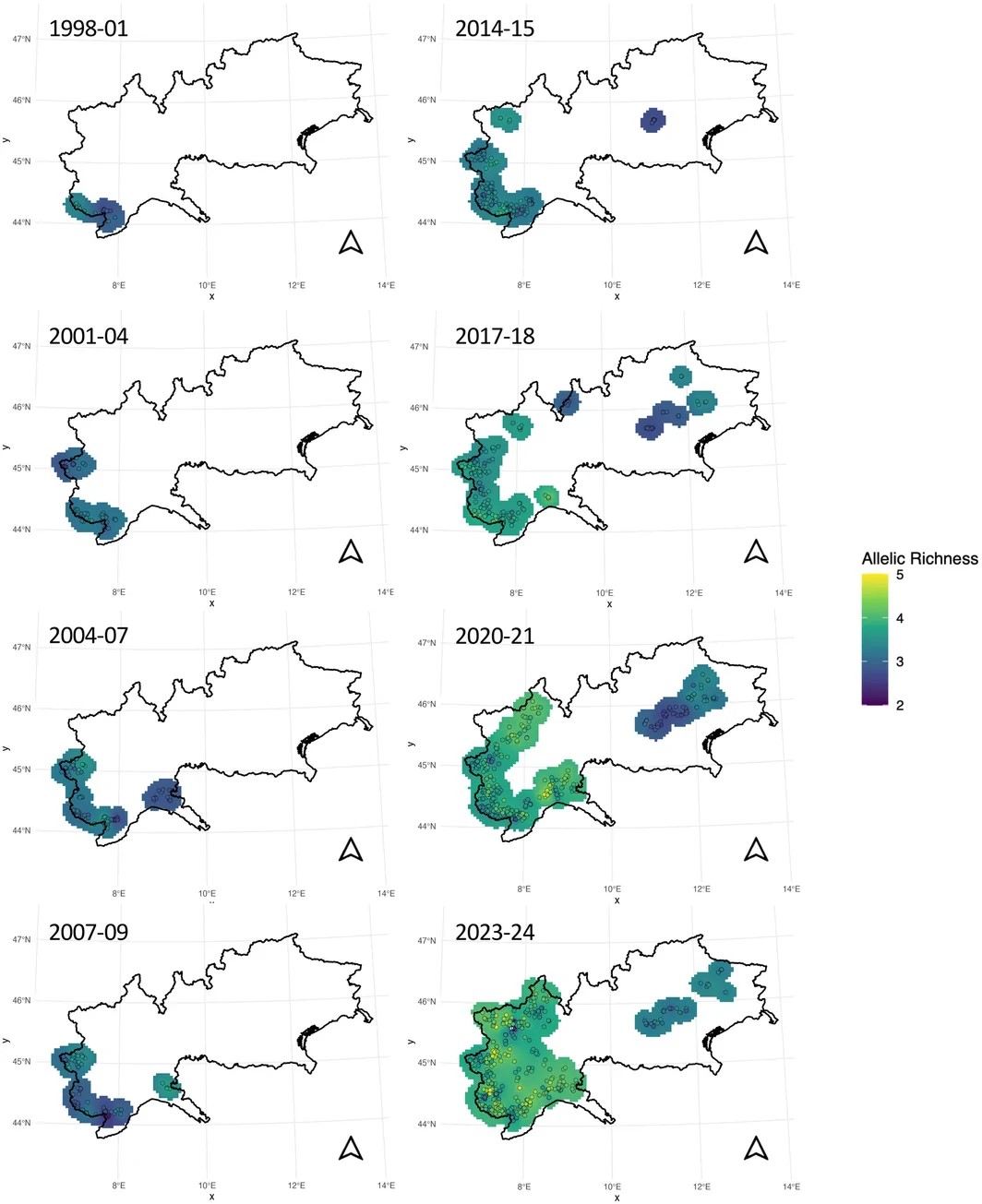
Spatially interpolated allelic richness (Ar) in wolves across the Italian Alps, from 1998 to 2024. Interpolated surfaces are clipped to 20 km buffers around sampling locations.

FIS revealed negative values in the years associated with founder effects (Figure 4). During the early expansion period (1998–2004), multilocus FIS values ranged from −0.35 to 0.30 across loci, with median values remaining below zero in all years. The lowest FIS was detected at the beginning of the recolonization, while the following years showed reduced variance among loci and FIS values approaching zero. In the late colonization period (2014–2023), patterns diverged between the two populations. While wolves in the west showed FIS values very close to zero, those in the east showed negative FIS values (−0.63 to 0.12) increasing over time. Pedigree reconstruction in the eastern Alps detected instances of mating between siblings (Marucco et al. 2018). Four newly established packs were founded by individuals descended from the initial founding pair. In two of these packs, the dominant breeding pair consisted of full siblings.

**FIGURE 4:**
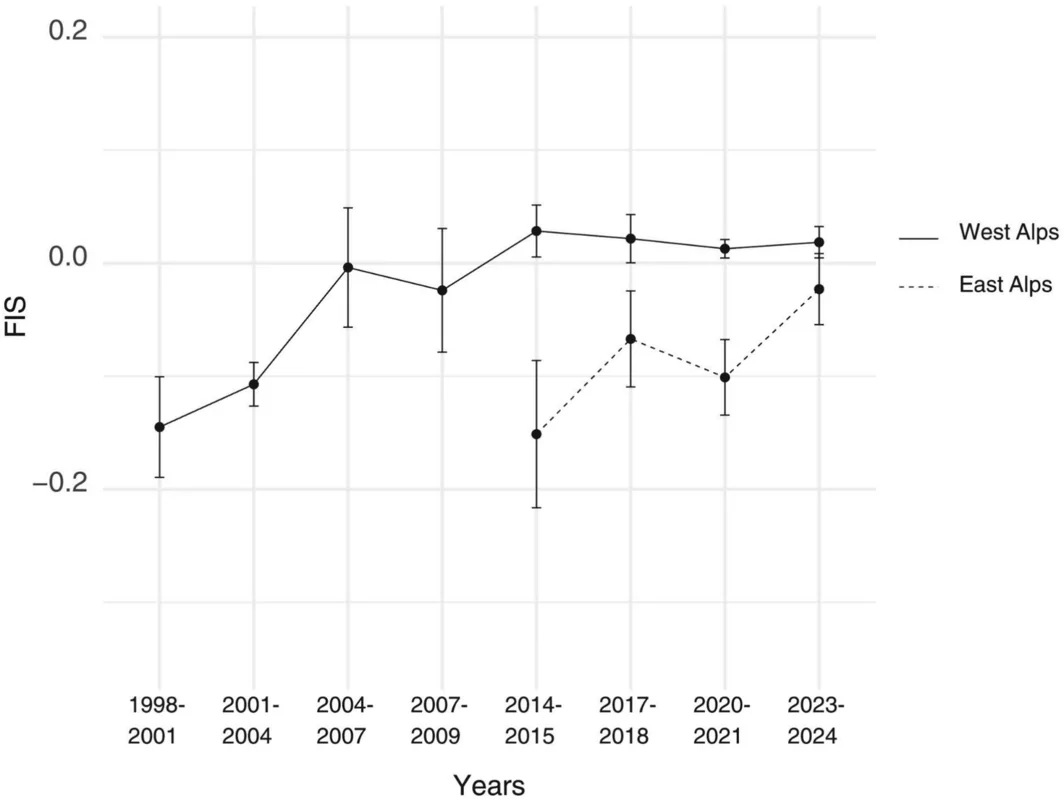
Mean FIS of the Italian Alpine wolf population over time (1998–2024). Points indicate the mean FIS per year, and vertical error bars represent the standard error of the mean. The solid line represents the western part of the population, and the dashed line represents the eastern part.

### 3.4 Genetic Connectivity of the Italian Alpine Wolf Population

Following the most recent stochastic colonization event in the Eastern Alps in 2014, our preliminary analysis revealed an asymmetric pattern of allele movement, with 85 alleles first detected in the west Alps and found at least one year later in the eastern Alps, while only 6 alleles first appeared in the east Alps before being discovered in the west. The western regions also showed a higher number of private alleles, with Pa_WEST_ = 59, across 15 loci (over a total of 158 alleles) and Pa_EAST_ = 14, across 8 loci (over a total of 113 alleles). The mean frequency of private alleles was p̄(1) = 0.0097. Based on Slatkin's private allele method (Barton and Slatkin 1986), the size‐corrected estimate of gene flow was Nm = 1.36. Contemporary estimates of gene flow confirmed the asymmetry inferred from allele‐spreading patterns but also showed very low migration rates, indicating that gene flow between eastern and western Alps remains limited. The estimated proportion of recent immigrants (or individuals with immigrant ancestry) in the eastern Alps originating from the western Alps (m) was consistently low over the last five years (m_2020‐21_ = 0.032 ± 0.013; m_2023‐24_ = 0.037 ± 0.018), while m in the opposite direction was almost absent (m_2020‐21_ = 0.018 ± 0.006; m_2023‐24_ = 0.006 ± 0.004). Within the broader western Alps, results from EEMS analyses revealed no resistance to gene flow (Figure 5), suggesting high genetic continuity within the region. Instead, within the eastern Alps, strong bands of resistance appeared to be present, despite some degree of enhanced connectivity. Consistent with these patterns, effective diversity rates showed pronounced spatial heterogeneity. In the western Alps, effective diversity was higher than the global average, with the highest values occurring in the area where the first individuals recolonizing from the Apennines established. In contrast, diversity in the eastern Alps was consistently lower than average, reaching its minimum in the region where the most recent founder event occurred.

**FIGURE 5:**
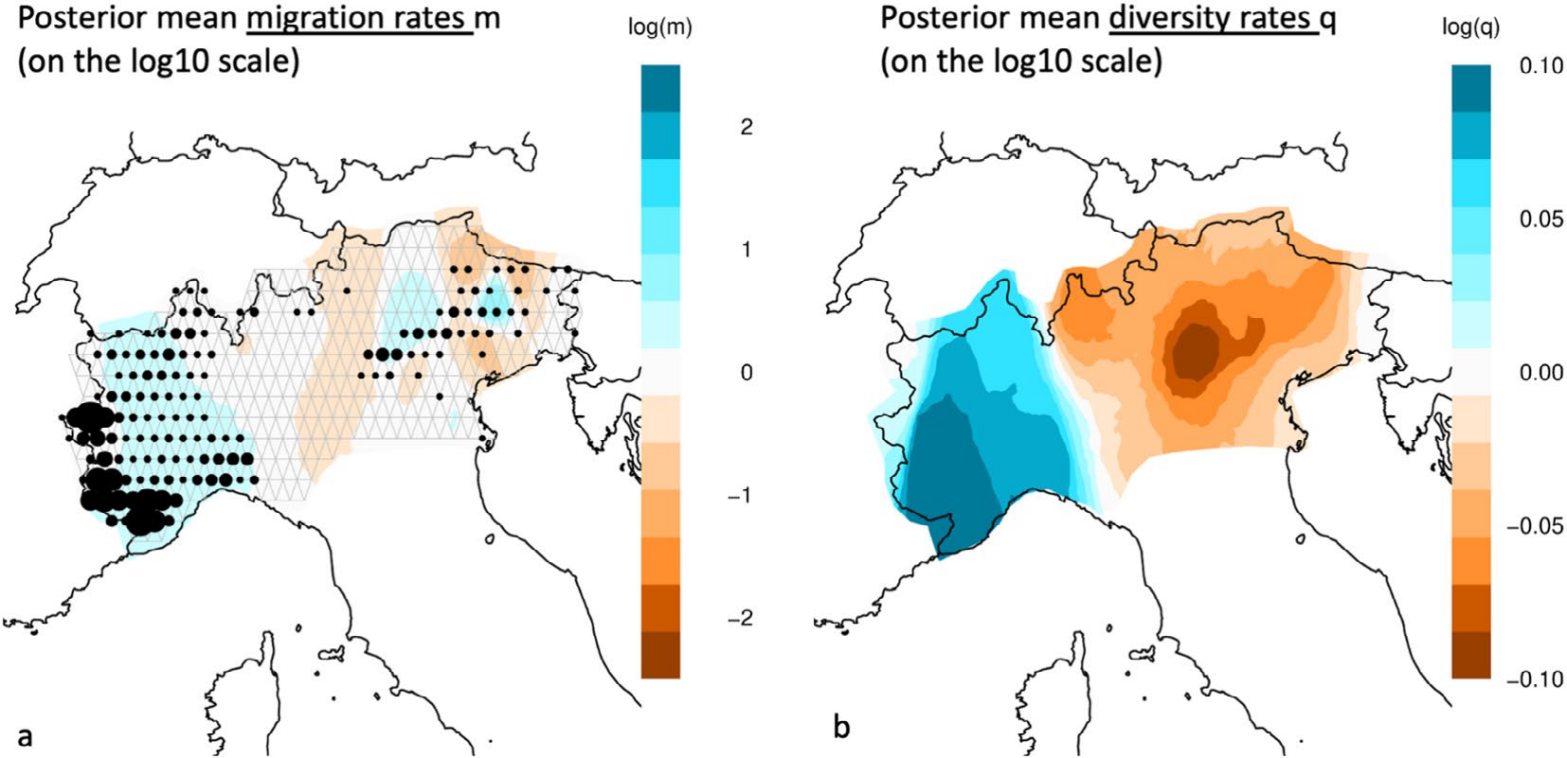
Effective migration surface (left) and effective diversity rates (right) for the Italian wolf population estimated using EEMS. On the left (a), blue shades indicate areas with higher migration rates than expected under isolation‐by‐distance, warm colours indicate lower migration rates than expected, and white areas represent expectations. On the right (b), blue indicates higher than average diversity and orange indicates lower than average diversity.

### 3.5 The Effective Population Size

Following the two previously identified genetic clusters, as determined by the population structure analysis and the very limited gene flow, we estimated *N*_e_ separately for the western and eastern Italian Alps. *N*_e_ estimates were highly consistent across the three temporal groupings (3‐year, 4‐year and 3‐year shifted by one year; details in Supplementary S1); therefore, we adopted a 3‐year generation length for reporting N̂_e_ trends through 2024 (Figure 6; Table S4). Estimates for the Italian Alpine population revealed a pronounced temporal increase in the western Alps, with all methods indicating very low N̂_e_ during the first cohort (1998–2001; SFrm: 13 CI 95% 7–30; SFnrm: 18 CI 95% 10–35; LDrm: 9.3 CI 95% 4.7–15.3; LDm: 15.4 CI 95% 9.5–25.7) and substantially higher values in 2020–2021 (SFrm: 118 CI 95% 91–153; SFnrm: 111 CI 95% 87–145; LDrm: 83.3 CI 95% 67.6–103.4; LDm: 165.9 CI 95% 135.1–205.2) and 2023–2024 (SFrm: 161 CI 95% 127–200; SFnrm: 152 CI 95% 123–193; LDrm: 122.2 CI 95% 97.7–154.6; LDm: 251.8 CI 95% 201.2–319.2), closely tracking the parallel increase in the number of packs.

**FIGURE 6:**
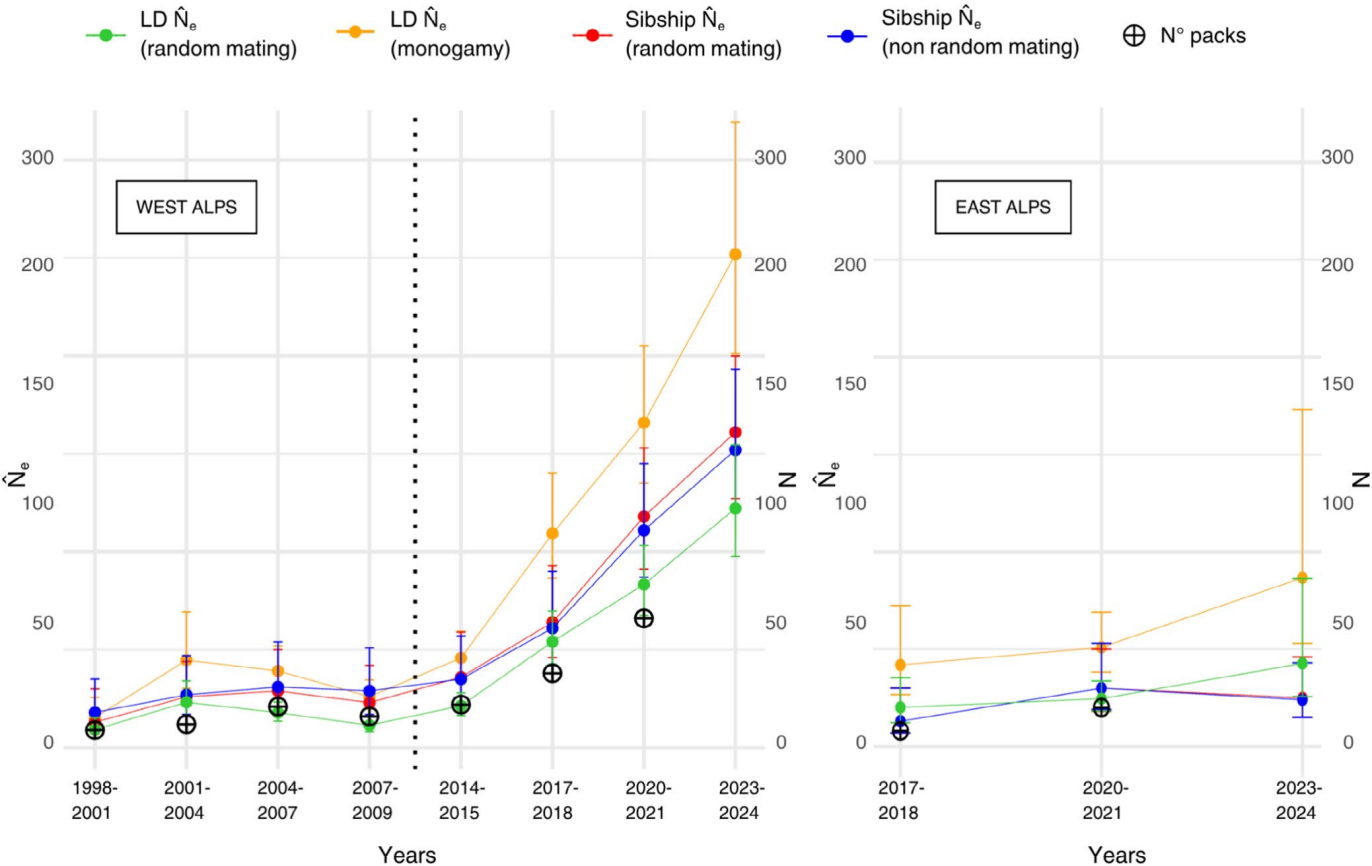
Estimated effective population size (N̂_e_) of the Italian Alpine wolf population from 1998 to 2024, shown separately for the western (left) and eastern (right) regions. Estimates include the linkage disequilibrium method assuming random mating (green), the linkage disequilibrium method assuming monogamy (yellow), the sibship method under random mating (red) and the sibship method under non‐random mating (blue). The number of packs for each year is shown as black circles. The vertical dashed line in the western plot indicates the 2010–2014 period during which systematic sampling was not conducted.

In the eastern Alps, N̂_e_ followed a similar trajectory, starting from very low values in 2017–2018 (SFrm and SFnrm: 13 CI 95% 7–30; LDrm: 20.1 CI 95% 12.3–35.4; LDm: 41.9 CI 95% 26.7–72.4), immediately after the founder event, increasing in 2020–2021 (SFrm: 30 CI95% 18–50; SFnrm: 30 CI 95% 19–53; LDrm: 24.6 CI 95% 18.2–33.7; LDm: 50.9 CI 95% 38.2–69.0), and then declining in 2023–2024 (SFrm: 25 CI 95% 15–46; SFnrm: 24 CI 95% 15–43; LDrm: 42.7 CI 95% 25.7–86.2; LDm: 86.7 CI 95% 53.0–172.9). Although still below the most recent *N*_e_ estimates in the western Alps, these values remain comparable to the first 15 years of population expansion in the west. The LDrm‐ and sibship‐based estimates of *N*_e_ were highly correlated across temporal cohorts (LDrm‐SFrm: Pearson's *r* = 0.972, *p* < 0.001; LDrm‐SFnrm: Pearson's *r* = 0.964, *p* < 0.001).

## 4 Discussion

Long‐term genetic monitoring of the Italian Alpine wolf population over the past three decades revealed that genetic recovery is underway across the Alps, but the process remains highly spatially structured. While the western Alps exhibit high genetic diversity and effective migration rates within the area, the eastern Alps still retain signatures of a recent isolated founder event. The two alpine areas showed very limited genetic connectivity. The persistence of high genetic differentiation between the western and eastern Alpine regions indicates that, despite demographic expansion in both areas, the time passed since recolonization from the Apennines and the Dinarics has not been sufficient to re‐establish genetic connectivity across the entire Alpine arc. The estimated effective population size has increased over time in the west, but the eastern Italian Alpine population is still in an early phase of genetic recovery.

By providing one of the most comprehensive datasets for the assessment of long‐term genetic dynamics on natural wolf recolonization in Europe, our study contributes to addressing the limited availability of extended genetic monitoring for recovering large carnivore populations, particularly in European landscapes (Liberg et al. 2005; Fabbri et al. 2007; Jansson et al. 2012; Caniglia et al. 2014; Granroth‐Wilding et al. 2017; Szewczyk et al. 2019; Jarausch et al. 2021). Natural wolf recolonization has typically proceeded through long‐distance dispersal events that allow a limited number of individuals to establish breeding packs far from source populations, followed by local population growth and secondary dispersal at shorter distances (Åkesson et al. 2022; Ražen et al. 2016; Mech and Boitani 2003). Genetic studies consistently show that these early colonization phases are marked by founder effects and reduced genetic diversity, whose magnitude depends on the number of founders, their relatedness and subsequent connectivity with source populations (Jarausch et al. 2021; Szewczyk et al. 2019; Granroth‐Wilding et al. 2017; Fabbri et al. 2007). We documented two temporally distinct founder events in the Italian Alpine wolf population, the first related to the initial recolonization of the western Alps in the late 1990s by wolves from the Apennine population, as already documented in Fabbri et al. 2007, followed by a second, stochastic colonization of the eastern Alps approximately a decade ago which we first documented. This second event accelerated the eastward expansion of the Alpine wolf population, while the central Italian Alps have been occupied more gradually. Colonization of this central sector was initially slow, partly because major pre‐Alpine lakes and the highly fragmented, human‐dominated landscape constrained connectivity. However, current spatial dynamics indicate the progressive occupation of the remaining areas, and further expansion across the central Alps is therefore expected over time (Boiani et al. 2026).

Our results suggest that both founder events were associated with reduced allelic richness; however, they also showed negative FIS values, likely resulting from the small population size of wolves during the first years of expansion. Although FIS is often referred to as an inbreeding coefficient, it measures departures from Hardy–Weinberg expectations under assumptions that are rarely met in small, recently founded natural populations. In particular, in populations with very small effective population sizes, stochastic sampling of alleles, differences in allele frequencies between breeding males and females, and constraints on random union of gametes in finite populations can generate negative FIS values even in the absence of assortative mating or outbreeding (Funk et al. 2016; Allendorf et al. 2022). Consequently, the observed heterozygote excess is consistent with the extremely small effective population size expected during the initial stages of recolonization. Accordingly, Fabbri et al. (2007) found a slight excess of heterozygotes in wolves in the western Alps following the bottleneck event. Evidence for relatedness and inbreeding following the founder events is instead provided by pedigree reconstruction presented in previous studies, which documented that breeding couples in the eastern Alps descended from the same founding pair (Marucco et al. 2018). Indeed, the second founder event, reported in the eastern Alps, showed stronger reduction in genetic diversity than that observed in the west, likely reflecting the extremely small number of initial colonizers (*n* = 2; Marucco et al. 2018; whereas previous estimates for the western Alps suggest 8–16 founding individuals; Fabbri et al. 2007). Similar patterns following founder events were also recorded in the German wolf population (Jarausch et al. 2021) and in Finland (Granroth‐Wilding et al. 2017). Following the founder events, wolves expanded in the western Alps across Italy, France and Switzerland, with substantial connectivity among these areas. Italy and France supported the vast majority of the Alpine population, while Austria represented a discontinuity for much of the study period, with few resident wolves (Marucco, Reinhardt, et al. 2023; Marucco, Boiani, et al. 2023). Reflecting the dynamics of wolf recolonization, the eastern Alps are connected to the north‐western Dinaric‐Balkan population through Slovenia, providing an important source of gene flow into the eastern portion of the Alpine range.

During the early phase of recolonization in the western region, the genetic structure inferred from our data reflected distinct lineage‐specific expansion from a limited number of founders. This pattern supports the conclusion that the Alpine wolf population was not established by a single breeding pair, but rather by multiple genetically unrelated colonizers (Fabbri et al. 2007). Early genetic differentiation reflected the pack‐based social structure characteristic of wolves. However, approximately a decade after the first establishment in the Alps, this structure shifted toward population‐level patterns. In the western Alps, two partially overlapping clusters remained detectable, while the eastern Alps formed a distinct genetic cluster. As recolonization progressed and the population expanded northward, genetic diversity increased through time. In fast growing populations, new alleles arising through mutations tend to persist as genetic drift is negligible (Allendorf et al. 2022). However, the period covered by our study makes mutation an unlikely explanation for the observed increase, which would require evolutionary timescales. Instead, the temporal increase in allelic richness is primarily explained by gene flow from the Apennine source population. Previous genetic studies showed that the western Alpine population originated through natural recolonization from the Italian Apennines and has since experienced persistent, predominantly unidirectional gene flow (Lucchini et al. 2002; Fabbri et al. 2007). Today, the contact zone between the Apennine and Alpine populations is continuously occupied by wolves (Marucco, Reinhardt, et al. 2023; Marucco, Boiani, et al. 2023), maintaining connectivity and allowing new alleles to enter the expanding Alpine population. We found that FIS stabilized following an initial phase of negative values, accompanied by the accumulation of private alleles, indicating ongoing immigration from the Apennine source population. High effective migration rates within the western Alps further support the progressive reduction of strong pack‐based genetic structure, suggesting that dispersals from the south successfully prevented long‐term genetic isolation of territorial units in this region. This outcome is particularly relevant in fragmented European landscapes, where connectivity can be easily compromised. Within the western Alps, effective diversity was higher across the whole study area. In particular, highest values were found in the south‐west region, which was previously independently identified by Marucco and McIntire (2010) as the primary source of dispersing wolves recolonizing the Alpine arc based on simulation analyses. Together, these independent lines of evidence confirm this area as the main origin of genetic differentiation within the Alps.

In contrast, the eastern Alps still show lower genetic diversity, genetic signatures of recent colonization and inbreeding events. Previous pedigree analyses showed the first packs in the eastern Alps to be founded by closely related individuals (Marucco et al. 2018). In particular, by 2017–2018, at least seven reproductive individuals in the eastern Alps were directly descended from the same founding pair. Spatially explicit analyses revealed pronounced resistance to gene flow within the eastern Alps, indicating that effective migration within the region is currently limited. The limited presence of wolves carrying the Dinaric‐Balkan haplotype also provides molecular evidence for still very limited admixture with the Eastern European population. However, allelic richness has increased since wolf establishment, suggesting an ongoing genetic recovery phase in this region, comparable to the first decade of recolonization in the western Alps, although gene flow from both the west and the Dinaric regions remains very limited.

Contemporary migration estimates indicate asymmetric gene flow, with a very low contribution of western‐Alpine immigrant ancestry to the eastern‐Alpine part of the population and almost no gene flow in the opposite direction. This effect is expected given the larger size of the western Alpine subpopulation, which is likely to contribute proportionally more dispersers under similar dispersal propensity and random dispersal distances. However, this extremely low west‐to‐east movement together with the persistence of a distinct eastern genetic cluster a decade after wolves' establishment in the region indicates that this time frame has not been sufficient for genetic reconnection between the two Alpine sectors, despite ongoing demographic expansion. A comparable situation has been reported in Poland, where three spatially structured wolf populations remain genetically distinct despite evidence of ongoing gene flow in this case (Szewczyk et al. 2019), highlighting that demographic recovery alone does not necessarily result in genetic homogenization at population‐level scales. Despite reduced connectivity and repeated founder events, neither Alpine sector showed persistent signals of departures from Hardy–Weinberg expectations. This suggests that wolf social structure, dispersal behaviour and occasional immigration from bordering populations can rapidly reduce the short‐term genetic consequences of isolation, as documented in both European (Caniglia et al. 2014) and North American wolf populations (vonholdt et al. 2008). This pattern raised the question on the number of individuals that have effectively contributed to reproduction through time, and whether demographic expansion has translated into relevant increases in effective population size (*N*_e_). Estimating *N*_e_ in wide‐ranging, socially structured species such as wolves requires careful consideration of spatial scale and mating system, as violations of model assumptions can substantially bias results (Mergeay et al. 2026). Linkage disequilibrium (LD)‐based estimators are sensitive to population structure because non‐random associations among alleles may arise not only from genetic drift in small populations, but also from spatial subdivision (Neel et al. 2013; Gilbert and Whitlock 2015), which is especially relevant in recolonizing populations, where spatial genetic structure is expected during early expansion phases. For this reason, we estimated *N*_e_ based on the genetic structure that emerged in this study. The sibship frequency (SF) method provides a complementary perspective on contemporary Ne by leveraging information on kinship structure. In small populations, a high proportion of full‐ and half‐sibling dyads is expected because relatively few breeders contribute offspring to each cohort, whereas the probability that two randomly sampled individuals are siblings declines as *N*_e_ increases. We found LD estimates in the Alpine population to be lower but comparable to SF estimates, particularly when population size was small. Previous studies showed that LD‐based *N*_e_ estimates are affected by age structure, with mixed‐age samples producing downwardly biased estimates, due to a two‐locus Wahlund effect that creates mixture LD when parents of different ages mate (Waples 2006). However, this downward bias tends to be smaller when the number of cohorts represented in the sample more closely approximates the generation length. The reproductive system is another important influence on LD‐based estimates. We included both random mating and monogamy assumptions, as wolf reproduction may deviate from random mating when dominated by a single pair over multiple breeding seasons. Under a predominantly monogamous system, assuming random mating would lead to downward‐biased estimates of *N*_e_. In the western Alps, N̂_e_ remained extremely low (N̂_e_ < 50) for the first 10–15 years following the founder event, before increasing to values exceeding 150 in the most recent years. These estimates are comparable to N̂_e_ values reported for other established European wolf populations when calculated at the subpopulation scale, which typically range from 100 to 300 depending on spatial delimitation and method (e.g., Germany, Spain; Mergeay et al. 2024). In contrast, N̂_e_ in the eastern Alps remains very low, showing only a slight increase over the last decade and reaching values over 50 in the most recent 2023/2024 period. However, this temporal trajectory closely mirrors that observed in the western Alps during the early stages of recolonization. This pattern is consistent with a more recent founder event and limited connectivity, suggesting that the eastern Italian Alpine population is still in an early phase of genetic recovery which needs attention from a conservation perspective. In this study, we conducted our estimates at the scale of the Italian Alpine population, as management and conservation measures are implemented at the national level and therefore require population‐specific assessments. However, genetic processes in wolves operate within transboundary metapopulations. The western Italian Alpine population is highly connected to the adjacent wolf population in France, and together they may constitute a single genetic unit. Consequently, *N*_e_ estimates derived from Italian samples alone should be interpreted as local effective population size (*N*_ex_; Fedorca et al. 2024). In our case, however, the temporal increase in allelic richness and the accumulation of private alleles within the western Italian Alps suggest that this area supports a high proportion of the genetic diversity of the broader western Alpine system, indicating that *N*_ex_ estimates based on Italian data alone may closely approximate *N*_e_ estimates of the entire genetic unit. In contrast, the eastern Italian Alpine population is smaller and more isolated. Here, *N*_ex_ is expected to be more sensitive to occasional immigration from neighbouring populations, as even low levels of successful gene flow may increase heterozygosity and, consequently, *N*_ex_ in this region. The most recent *N*_ex_ estimates for the western Alps exceed the short‐term threshold of ≥ 50, commonly cited as necessary to avoid immediate inbreeding depression (Jamieson and Allendorf 2012; Frankham et al. 2013) after approximately a decade during which the estimated *N*_ex_ remained below this value. In contrast, most recent *N*_ex_ estimates for the eastern Alps remain below this threshold, indicating that this region warrants particular attention and continued monitoring. From a long‐term conservation perspective, *N*_ex_ still falls below the target of ≥ 500 proposed for maintaining adaptive potential (Franklin 1980; Lande and Barrowclough 1987), now incorporated as an indicator in the CBD Global Biodiversity Framework (Frankham 2022; Hoban et al. 2023). However, these commonly used guidelines for effective population size are intended to be considered at the metapopulation scale. Since our analyses did not include genetic samples outside the Italian border, and the Apennine population may also contribute to the effective size of the broader genetic system, we advise caution in interpreting these estimates as measures of metapopulation *N*_e_. Nevertheless, these values remain highly relevant for assessing the genetic status of the Italian Alpine wolf population and for guiding conservation strategies aimed at maintaining connectivity with neighbouring populations, thereby supporting its long‐term genetic diversity and adaptive potential.

Recent work in European landscapes has shown that pack number can be a reasonable proxy for estimated *N*_e_ in wolves when populations are analysed at appropriate spatial scales (Mergeay et al. 2024). Our results support this conclusion for the Italian Alps, particularly with respect to LD‐based *N*_e_ estimate assuming random mating, especially during the early phase of population expansion and when spatial population structure was accounted for by analysing the eastern and western subpopulations separately. However, extrapolation to the entire wolf Alpine population should be made with caution, as we found stronger support for the relationship at lower N̂_e_ values and packs in neighbouring areas extending outside Italy's borders were not accounted for.

## 5 Conclusion

In this study, following three decades of natural wolf expansion in the Alps, we found persistent genetic distinctiveness between the western and eastern Italian Alpine regions, despite ongoing demographic expansion. The western Alps currently act as a primary source of genetic diversity, with higher allelic richness, higher within‐region migration rates and a detectable increase in the estimated effective population size. The eastern Alps still exhibit lower genetic diversity, smaller N̂_e_, signatures of recent founder events and limited connectivity, suggesting the importance of maintaining close monitoring in this region. Although gene flow within the western Alps appears sufficient to prevent detectable inbreeding, the recent stabilization of N̂_e_ in 2023/2024 suggests that continued genetic monitoring is still needed. Our results demonstrate that demographic recovery alone does not necessarily translate into genetic reconnection or long‐term evolutionary viability. Long‐term genetic monitoring provides essential insights that complement demographic data and is critical for informing conservation strategies aimed at maintaining connectivity, preserving adaptive potential, and ensuring the long‐term viability of recolonizing large carnivore populations, especially when these inhabit fragmented landscapes.

## Author Contributions

**Francesca Marucco:** designed research; performed research; writing – original draft, writing – review and editing; supervision. **Francesca Rolle:** analysed data; writing – original draft; writing – review and editing. **Elisa Avanzinelli:** performed research; writing – review and editing. **Kristine L. Pilgrim:** laboratory analysis; writing – review and editing; supervision. **Michael K. Schwartz:** laboratory analysis; writing – review and editing; supervision.

## Funding

This work was supported by LIFE programme, LIFE WolfAlps 2013‐2018 LIFE12 NAT/IT/000807, LIFE WolfAlps EU 2020‐2024 LIFE18 NAT/IT/000972.

## Disclosure

Benefit‐sharing section: Our group is committed to international scientific partnerships; the results of research have been shared with the provider communities and the broader scientific community; and the research addresses a priority concern–long‐term genetic monitoring to inform conservation strategies aimed at maintaining connectivity, preserving adaptive potential and ensuring the long‐term viability of recolonizing large carnivore populations, especially when these inhabit fragmented landscapes.

## Ethics Statement

This study was conducted using non‐invasive methods to monitor the Italian Alpine wolf population from 1998 to 2024. Data collection involved the systematic gathering of indirect signs. At no point were wolves captured, handled, or otherwise disturbed during the research process. Given the non‐invasive nature of the study, no specific animal handling licences or permits were required. This approach aligns with ethical guidelines for wildlife research, ensuring minimal impact on the animals and their habitats.

## Conflicts of Interest

The authors declare no conflicts of interest.

## Supporting information


**Supplementary S1.** Methods and results of effective population size (N̂e) estimation for the Italian Alpine wolf population, 1998–2024. Methods and results of N̂e estimation using Linkage Disequilibrium and Sibship Frequency approaches, including sensitivity tests on generation length and starting year (Tables S1–S3) and estimates by cohort for the western and eastern Alps (Table S4).
**Table S1:** Estimates of N̂e of the Italian Alpine wolf population, sampled as four cohorts of three years each between 1998 and 2010. SF rm.: Sibship N̂e assuming random mating; SF nrm: Sibship N̂e assuming non random mating; LD: Linkage Disequilibrium N̂e.
**Table S2:** Estimates of N̂e of the Italian Alpine wolf population, sampled as three cohorts of four years each between 1998 and 2009. SF rm.: Sibship N̂e assuming random mating; SF nrm: Sibship N̂e assuming non random mating; LD: Linkage Disequilibrium N̂e.
**Table S3:** Estimates of N̂e of the Italian Alpine wolf population, sampled as four cohorts of three years each between 1999 and 2010. SF rm.: Sibship N̂e assuming random mating; SF nrm: Sibship N̂e assuming non random mating; LD: Linkage Disequilibrium N̂e.
**Table S4:** Estimates of effective population size (N̂e) for the Italian Alpine wolf population, calculated from three‐year cohorts spanning 1998–2024, with separate analyses for the western and eastern Alps. SF rm.: Sibship N̂e assuming random mating; SF nrm: Sibship N̂e assuming non random mating; LD rm.: Linkage Disequilibrium N̂e assuming random mating; LD m: Linkage Disequilibrium N̂e assuming monogamy The number of packs observed in each time period is also reported.

## Data Availability

The data that support the findings of this study are openly available in Zenodo at https://zenodo.org/records/22709950.

## References

[mec70639-bib-0001] Åkesson, M. , Ø. Flagstad , J. Aspi , et al. 2022. “Genetic Signature of Immigrants and Their Effect on Genetic Diversity in the Recently Established Scandinavian Wolf Population.” Conservation Genetics 23, no. 2: 359–373. 10.1007/s10592-021-01423-5.

[mec70639-bib-0002] Allendorf, F. , W. C. Funk , S. Aitken , M. Byrne , G. Luikart , and A. Antunes . 2022. Conservation and the Genomics of Populations. 3rd ed. Oxford University Press. 10.1093/oso/9780198856566.001.0001. PMC9753819 36540641

[mec70639-bib-0003] Aspi, J. , E. Roininen , M. Ruokonen , I. Kojola , and C. Vilà . 2006. “Genetic Diversity, Population Structure, Effective Population Size and Demographic History of the Finnish Wolf Population.” Molecular Ecology 15: 1561–1576. 10.1111/j.1365-294X.2006.02877.x. 16629811

[mec70639-bib-0086] Barton, N. H. , and M. Slatkin . 1986. “A Quasi‐Equilibrium Theory of the Distribution of Rare Alleles in a Subdivided Population.” Heredity 56, no. 3: 409–415. 10.1038/hdy.1986.63. 3733460

[mec70639-bib-0004] Battilani, D. , R. Gargiulo , R. Caniglia , et al. 2025. “Beyond Population Size: Whole‐Genome Data Reveal Bottleneck Legacies in the Peninsular Italian Wolf.” Journal of Heredity 116, no. 1: 10–23. 10.1093/jhered/esae041. PMC11700593 39189963

[mec70639-bib-0087] Boiani, M. V. , P. Dupont , R. Bischof , et al. 2026. “Changing Patterns of Colonisation and Persistence During the Wolf Recolonisation of the Human‐Dominated Italian Alpine Region.” Ecography 7: e08044. 10.1002/ecog.08044.

[mec70639-bib-0005] Caniglia, R. , E. Fabbri , M. Galaverni , P. Milanesi , and E. Randi . 2014. “Noninvasive Sampling and Genetic Variability, Pack Structure, and Dynamics in an Expanding Wolf Population.” Journal of Mammalogy 95, no. 1: 41–59. 10.1644/13-MAMM-A-039.

[mec70639-bib-0006] Caniglia, R. , E. Fabbri , M. Konec , et al. 2021. “International Genetic Protocols for Cost‐Effective Monitoring of the Alpine Wolf Population and Detection of Hybridization.” Report for LIFE WolfAlps EU project LIFE18 NAT/IT/000972 Action A5‐A6.

[mec70639-bib-0007] Chapron, G. , P. Kaczensky , J. D. Linnell , et al. 2014. “Recovery of Large Carnivores in Europe's Modern Human‐Dominated Landscapes.” Science 346, no. 6216: 1517–1519. 10.1126/science.1257553. 25525247

[mec70639-bib-0008] Day, C. C. , E. L. Landguth , M. A. Sawaya , et al. 2024. “Genetic Connectivity of Wolverines in Western North America.” Scientific Reports 14, no. 1: 28248. 10.1038/s41598-024-77956-9. PMC11568290 39548133

[mec70639-bib-0009] De Barba, M. , L. P. Waits , E. O. Garton , et al. 2010. “The Power of Genetic Monitoring for Studying Demography, Ecology and Genetics of a Reintroduced Brown Bear Population.” Molecular Ecology 19, no. 18: 3938–3951. 10.1111/j.1365-294X.2010.04791.x. 20735733

[mec70639-bib-0010] Di Bernardi, C. , G. Chapron , P. Kaczensky , et al. 2025. “Continuing Recovery of Wolves in Europe.” PLOS Sustainability and Transformation 4, no. 2: e0000158. 10.1371/journal.pstr.0000158.

[mec70639-bib-0011] Do, C. , R. S. Waples , D. Peel , G. M. Macbeth , B. J. Tillett , and J. R. Ovenden . 2014. “NeEstimator V2: Re‐Implementation of Software for the Estimation of Contemporary Effective Population Size (Ne) From Genetic Data.” Molecular Ecology Resources 14: 209–214. 10.1111/1755-0998.12157. 23992227

[mec70639-bib-0012] Earl, D. A. , and B. M. vonHoldt . 2012. “STRUCTURE HARVESTER: A Website and Program for Visualizing STRUCTURE Output and Implementing the Evanno Method.” Conservation Genetics Resources 4: 359–361. 10.1007/s12686-011-9548-7.

[mec70639-bib-0013] Evanno, G. , S. Regnaut , and J. Goudet . 2005. “Detecting the Number of Clusters of Individuals Using the Software Structure: A Simulation Study.” Molecular Ecology 14: 2611–2620. 10.1111/j.1365-294X.2005.02553.x. 15969739

[mec70639-bib-0014] Fabbri, E. , C. Miquel , V. Lucchini , et al. 2007. “From the Apennines to the Alps: Colonization Genetics of the Naturally Expanding Italian Wolf ( *Canis lupus* ) Population.” Molecular Ecology 16, no. 8: 1661–1671. 10.1111/j.1365-294X.2007.03262.x. 17402981

[mec70639-bib-0015] Fedorca, A. , J. Mergeay , A. O. Akinyele , et al. 2024. “Dealing With the Complexity of Effective Population Size in Conservation Practice.” Evolutionary Applications 17: e70031. 10.1111/eva.70031. PMC11645448 39679127

[mec70639-bib-0016] Fontsere, C. , S. A. Speak , A. J. Caven , et al. 2025. “Persistent Genomic Erosion in Whooping Cranes Despite Demographic Recovery.” Molecular Ecology 34, no. 23: e70088. 10.1111/mec.70088. PMC12684310 40856093

[mec70639-bib-0017] Fraik, A. K. , K. L. Pilgrim , C. E. Mosby , et al. 2025. “The Genetic Implications of Translocations on Fisher ( *Pekania pennanti* ) Populations in Montana and Idaho.” Journal of Mammalogy 106, no. 6: 1311–1323. 10.1093/jmammal/gyaf065.

[mec70639-bib-0018] Frankham, R. 2022. “Evaluation of Proposed Genetic Goals and Targets for the Convention on Biological Diversity.” Conservation Genetics 23, no. 5: 865–870. 10.1007/s10592-022-01459-1.

[mec70639-bib-0019] Frankham, R. , B. W. Brook , C. J. Bradshaw , L. W. Traill , and D. Spielman . 2013. “50/500 Rule and Minimum Viable Populations: Response to Jamieson and Allendorf.” Trends in Ecology & Evolution 28, no. 4: 187–188. 10.1016/j.tree.2013.01.002. 23369407

[mec70639-bib-0020] Franklin, I. R. 1980. “Evolutionary Change in Small Populations.” In Conservation Biology: An Evolutionary‐Ecological Perspective, edited by M. E. Soulé and B. A. Wilcox , 135–150. Sinauer.

[mec70639-bib-0021] Funk, W. C. , R. E. Lovich , P. A. Hohenlohe , et al. 2016. “Adaptive Divergence Despite Strong Genetic Drift: Genomic Analysis of the Evolutionary Mechanisms Causing Genetic Differentiation in the Island Fox ( *Urocyon littoralis* ).” Molecular Ecology 25: 2176–2194. 10.1111/mec.13605. PMC4877267 26992010

[mec70639-bib-0022] Gilbert, K. J. , and M. C. Whitlock . 2015. “Evaluating Methods for Estimating Local Effective Population Size With and Without Migration.” Evolution 69, no. 8: 2154–2166. 10.1111/evo.12713. 26118738

[mec70639-bib-0023] Goudet, J. 2005. “Hierfstat, a Package for r to Compute and Test Hierarchical F‐Statistics.” Molecular Ecology Notes 5: 184–186. 10.1111/j.1471-8286.2004.00828.x.

[mec70639-bib-0024] Granroth‐Wilding, H. , C. Primmer , M. Lindqvist , et al. 2017. “Non‐Invasive Genetic Monitoring Involving Citizen Science Enables Reconstruction of Current Pack Dynamics in a Re‐Establishing Wolf Population.” BMC Ecology 17: 44. 10.1186/s12898-017-0154-8. PMC5738207 29258497

[mec70639-bib-0025] Hendricks, S. A. , R. M. Schweizer , R. J. Harrigan , et al. 2019. “Natural Re‐Colonization and Admixture of Wolves ( *Canis lupus* ) in the US Pacific Northwest: Challenges for the Protection and Management of Rare and Endangered Taxa.” Heredity 122: 133–149. 10.1038/s41437-018-0094-x. PMC6327037 29880893

[mec70639-bib-0026] Hill, W. G. 1981. “Estimation of Effective Population Size From Data on Linkage Disequilibrium.” Genetical Research 38, no. 3: 209–216. 10.1017/S0016672300020553.

[mec70639-bib-0027] Hoban, S. , M. W. Bruford , J. M. da Silva , et al. 2023. “Genetic Diversity Goals and Targets Have Improved, but Remain Insufficient for Clear Implementation of the Post‐2020 Global Biodiversity Framework.” Conservation Genetics 24: 181–191. 10.1007/s10592-022-01492-0. PMC9841145 36683963

[mec70639-bib-0028] Hoban, S. , M. W. Bruford , W. C. Funk , et al. 2021. “Global Commitments to Conserving and Monitoring Genetic Diversity Are Now Necessary and Feasible.” Bioscience 71, no. 9: 964–976. 10.1093/BIOSCI/BIAB054. PMC8407967 34475806

[mec70639-bib-0029] Hohenlohe, P. A. , W. C. Funk , and O. P. Rajora . 2021. “Population Genomics for Wildlife Conservation and Management.” Molecular Ecology 30, no. 1: 62–82. 10.1111/mec.15720. PMC7894518 33145846

[mec70639-bib-0030] Jamieson, I. G. , and F. W. Allendorf . 2012. “How Does the 50/500 Rule Apply to MVPs?” Trends in Ecology & Evolution 27, no. 10: 578–584. 10.1016/j.tree.2012.07.001. 22868005

[mec70639-bib-0031] Jansson, E. , M. Ruokonen , I. Kojola , and J. Aspi . 2012. “Rise and Fall of a Wolf Population: Genetic Diversity and Structure During Recovery, Rapid Expansion and Drastic Decline.” Molecular Ecology 21, no. 21: 5178–5193. 10.1111/mec.12010. 22978518

[mec70639-bib-0032] Jarausch, A. , V. Harms , G. Kluth , I. Reinhardt , and C. Nowak . 2021. “How the West Was Won: Genetic Reconstruction of Rapid Wolf Recolonization Into Germany's Anthropogenic Landscapes.” Heredity 127, no. 1: 92–106. 10.1038/s41437-021-00429-6. PMC8249462 33846578

[mec70639-bib-0033] Jensen, E. L. , D. L. Edwards , R. C. Garrick , et al. 2018. “Population Genomics Through Time Provides Insights Into the Consequences of Decline and Rapid Demographic Recovery Through Head‐Starting in a Galapagos Giant Tortoise.” Evolutionary Applications 11, no. 10: 1811–1821. 10.1111/EVA.12682. PMC6231475 30459831

[mec70639-bib-0034] Jones, O. R. , and J. Wang . 2010. “COLONY: A Program for Parentage and Sibship Inference From Multilocus Genotype Data.” Molecular Ecology Resources 10: 551–555. 10.1111/j.1755-0998.2009.02787.x. 21565056

[mec70639-bib-0035] Keller, L. F. , I. Biebach , S. R. Ewing , and P. E. Hoeck . 2012. “The Genetics of Reintroductions: Inbreeding and Genetic Drift.” In Reintroduction Biology, edited by J. G. Ewen , D. P. Armstrong , K. A. Parker , and P. J. Seddon , 360–394. Blackwell Publishing Ltd. 10.1002/9781444355833.ch11.

[mec70639-bib-0036] Kuijper, D. P. J. , T. A. Diserens , E. Say‐Sallaz , et al. 2024. “Wolves Recolonize Novel Ecosystems Leading to Novel Interactions.” Journal of Applied Ecology 61: 906–921. 10.1111/1365-2664.14602.

[mec70639-bib-0037] Lande, R. , and G. F. Barrowclough . 1987. “Effective Population Size, Genetic Variation, and Their Use in Population Management.” In Viable Populations for Conservation, edited by M. E. Soulé . Cambridge University Press.

[mec70639-bib-0038] Leroy, G. , E. L. Carroll , M. W. Bruford , et al. 2018. “Next‐Generation Metrics for Monitoring Genetic Erosion Within Populations of Conservation Concern.” Evolutionary Applications 11, no. 7: 1066–1083. 10.1111/EVA.12564. PMC6050182 30026798

[mec70639-bib-0039] Liberg, O. , H. Andrén , H. C. Pedersen , et al. 2005. “Severe Inbreeding Depression in a Wild Wolf ( *Canis lupus* ) Population.” Biological Letters 1: 17–20. 10.1098/rsbl.2004.0266. PMC1629062 17148117

[mec70639-bib-0040] Lowe, W. H. , and F. W. Allendorf . 2010. “What Can Genetics Tell Us About Population Connectivity?” Molecular Ecology 19, no. 15: 3038–3051. 10.1111/J.1365-294X.2010.04688.X. 20618697

[mec70639-bib-0041] Lucchini, V. , E. Fabbri , F. Marucco , S. Ricci , L. Boitani , and E. Randi . 2002. “Noninvasive Molecular Tracking of Colonizing Wolf ( *Canis lupus* ) Packs in the Western Italian Alps.” Molecular Ecology 11, no. 5: 857–868. 10.1046/j.1365-294X.2002.01489.x. 11975702

[mec70639-bib-0042] Lucchini, V. , A. Galov , and E. Randi . 2004. “Evidence of Genetic Distinction and Long‐Term Population Decline in Wolves ( *Canis lupus* ) in the Italian Apennines.” Molecular Ecology 13: 523–536. 10.1046/j.1365-294X.2004.02077.x. 14871358

[mec70639-bib-0043] Luikart, G. , T. Antao , B. K. Hand , et al. 2021. “Detecting Population Declines via Monitoring the Effective Number of Breeders (Nb).” Molecular Ecology Resources 21, no. 2: 379–393. 10.1111/1755-0998.13251. 32881365

[mec70639-bib-0044] Luikart, G. , P. R. England , D. Tallmon , S. Jordan , and P. Taberlet . 2003. “The Power and Promise of Population Genomics: From Genotyping to Genome Typing.” Nature Reviews Genetics 4, no. 12: 981–994. 10.1038/NRG1226;KWRD. 14631358

[mec70639-bib-0045] Marucco, F. , E. Avanzinelli , B. Bassano , et al. 2018. “La Popolazione di Lupo Sulle Alpi Italiane 2014–2018.” Relazione Tecnica, Progetto LIFE 12 NAT/IT/00080 WOLFALPS – Azione A4 e D1.

[mec70639-bib-0046] Marucco, F. , E. Avanzinelli , M. V. Boiani , et al. 2022. “La Popolazione di Lupo Nelle Regioni Alpine Italiane 2020–2021.” Relazione Tecnica Dell'attività di Monitoraggio Nazionale Nell'ambito Del Piano di Azione Del Lupo ai Sensi Della Convenzione ISPRAMITE e Nell'ambito Del Progetto LIFE 18 NAT/IT/000972 WOLFALPS EU.

[mec70639-bib-0047] Marucco, F. , M. V. Boiani , P. Dupont , et al. 2023. “A Multidisciplinary Approach to Estimating Wolf Population Size for Long‐Term Conservation.” Conservation Biology 37, no. 6: e14132. 10.1111/COBI.14132. 37259636

[mec70639-bib-0049] Marucco, F. , L. Boitani , D. H. Pletscher , and M. K. Schwartz . 2011. “Bridging the Gaps Between Non‐Invasive Genetic Sampling and Population Parameter Estimation.” European Journal of Wildlife Research 57: 1–13. 10.1007/s10344-010-0477-7.

[mec70639-bib-0050] Marucco, F. , V. La Morgia , P. Aragno , et al. 2020. “Linee Guida e Protocolli per il Monitoraggio Nazionale Del Lupo in Italia.” Realizzate Nell'ambito Della Convenzione ISPRA‐Ministero Dell'ambiente e Della Tutela del Territorio e Del Mare Per “Attività di Monitoraggio Nazionale Nell'ambito Del Piano di Azione Del Lupo”.

[mec70639-bib-0051] Marucco, F. , and E. J. B. McIntire . 2010. “Predicting Spatio‐Temporal Recolonization of Large Carnivore Populations and Livestock Depredation Risk: Wolves in the Italian Alps.” Journal of Applied Ecology 47: 789–798. 10.1111/j.1365-2664.2010.01831.x.

[mec70639-bib-0052] Marucco, F. , K. L. Pilgrim , E. Avanzinelli , M. K. Schwartz , and L. Rossi . 2022. “Wolf Dispersal Patterns in the Italian Alps and Implications for Wildlife Diseases Spreading.” Animals 12, no. 10: 1260. 10.3390/ANI12101260. PMC9137635 35625106

[mec70639-bib-0053] Marucco, F. , D. H. Pletscher , L. Boitani , M. K. Schwartz , K. L. Pilgrim , and J. D. Lebreton . 2009. “Wolf Survival and Population Trend Using Non‐Invasive Capture–Recapture Techniques in the Western Alps.” Journal of Applied Ecology 46, no. 5: 1003–1010. 10.1111/j.1365-2664.2009.01696.x.

[mec70639-bib-0054] Marucco, F. , I. Reinhardt , E. Avanzinelli , et al. 2023. “Transboundary Monitoring of the Wolf Alpine Population Over 21 Years and Seven Countries.” Animals 13, no. 22: 3551. 10.3390/ani13223551. PMC10668717 38003168

[mec70639-bib-0055] McKelvey, K. S. , and M. K. Schwartz . 2005. “Dropout: A Program to Identify Problem Loci and Samples for Noninvasive Genetic Samples in a Capture‐Mark‐Recapture Framework.” Molecular Ecology Notes 5: 716–718. 10.1111/j.1471-8286.2005.01038.x.

[mec70639-bib-0056] Mech, L. D. , and L. Boitani . 2003. “Wolf Social Ecology.” In Wolves: Behaviour, Ecology and Conservation, edited by L. D. Mech and L. Boitani , 1–34. University of Chicago Press.

[mec70639-bib-0057] Meirmans, P. G. 2014. “Nonconvergence in Bayesian Estimation of Migration Rates.” Molecular Ecology Resources 14: 726–733. 10.1111/1755-0998.12216. 24373147

[mec70639-bib-0058] Mergeay, J. , R. Gargiulo , Y. Moodley , and I.‐R. Russo . 2026. “The Importance of Effective Population Size in Conservation and Biodiversity Monitoring.” Evolutionary Applications 19: e70196. 10.1111/eva.70196. PMC12811847 41550135

[mec70639-bib-0059] Mergeay, J. , S. Smet , S. Collet , et al. 2024. “Estimating the Effective Size of European Wolf Populations.” Evolutionary Applications 17: e70021. 10.1111/eva.70021. PMC11494449 39439436

[mec70639-bib-0060] Neel, M. C. , K. McKelvey , N. Ryman , et al. 2013. “Estimation of Effective Population Size in Continuously Distributed Populations: There Goes the Neighborhood.” Heredity 111, no. 3: 189–199. 10.1038/hdy.2013.37. PMC3746818 23652561

[mec70639-bib-0061] Pazhenkova, E. , M. Bartol , B. Boljte , et al. 2025. “Genetic Rescue of the Dinaric Lynx Population: Insights for Conservation From Genetic Monitoring and Individual‐Based Modelling.” Evolutionary Applications 18, no. 1: e70045. 10.1111/eva.70045. PMC11718419 39802318

[mec70639-bib-0062] Pérez‐Sorribes, L. , P. Villar‐Yanez , L. Smeds , and J. Mergeay . 2024. “Comparing Genetic ne Reconstructions Over Time With Long‐Time Wolf Monitoring Data in Two Populations.” Evolutionary Applications 17: e70022. 10.1111/eva.70022. PMC11486914 39430439

[mec70639-bib-0063] Petkova, D. , J. Novembre , and M. Stephens . 2016. “Visualizing Spatial Population Structure With Estimated Effective Migration Surfaces.” Nature Genetics 48: 94–100. 10.1038/ng.3464. PMC4696895 26642242

[mec70639-bib-0064] Pritchard, J. K. , M. Stephens , and P. Donnelly . 2000. “Inference of Population Structure Using Multilocus Genotype Data.” Genetics 155, no. 2: 945–959. 10.1093/genetics/155.2.945. PMC1461096 10835412

[mec70639-bib-0065] R Core Team . 2025. “R: A Language and Environment for Statistical Computing.” R Foundation for Statistical Computing, Vienna, Austria. https://www.R‐project.org/.

[mec70639-bib-0066] Rambaut, A. , M. A. Suchard , D. Xie , and A. J. Drummond . 2014. “Tracer v1.6.” http://beast.bio.ed.ac.uk/Tracer.

[mec70639-bib-0067] Ražen, N. , A. Brugnoli , C. Castagna , et al. 2016. “Long‐Distance Dispersal Connects Dinaric‐Balkan and Alpine Grey Wolf ( *Canis lupus* ) Populations.” European Journal of Wildlife Research 62: 137–142. 10.1007/s10344-015-0971-z.

[mec70639-bib-0068] Reed, D. H. , and R. Frankham . 2003. “Correlation Between Fitness and Genetic Diversity.” Conservation Biology 17, no. 1: 230–237. 10.1046/j.1523-1739.2003.01236.x.

[mec70639-bib-0069] Rousset, F. 2008. “genepop'007: A Complete Re‐Implementation of the Genepop Software for Windows and Linux.” Molecular Ecology Resources 8: 103–106. 10.1111/j.1471-8286.2007.01931.x. 21585727

[mec70639-bib-0070] Sastre, N. , C. Vilà , M. Salinas , et al. 2011. “Signatures of Demographic Bottlenecks in European Wolf Populations.” Conservation Genetics 12: 701–712. 10.1007/s10592-010-0177-6.

[mec70639-bib-0071] Schwartz, M. K. , S. A. Cushman , K. S. McKelvey , J. Hayden , and C. Engkjer . 2006. “Detecting Genotyping Errors and Describing American Black Bear Movement in Northern Idaho.” Ursus 17, no. 2: 138–148. 10.2192/1537-6176(2006)17[138:DGEADA]2.0.CO;2.

[mec70639-bib-0072] Schwartz, M. K. , G. Luikart , and R. S. Waples . 2007. “Genetic Monitoring as a Promising Tool for Conservation and Management.” Trends in Ecology & Evolution 22, no. 1: 25–33. 10.1016/j.tree.2006.08.009. 16962204

[mec70639-bib-0073] Shaw, R. E. , K. A. Farquharson , M. W. Bruford , et al. 2025. “Global Meta‐Analysis Shows Action Is Needed to Halt Genetic Diversity Loss.” Nature 638: 704–710. 10.1038/s41586-024-08458-x. PMC11839457 39880948

[mec70639-bib-0074] Szewczyk, M. , S. Nowak , N. Niedźwiecka , et al. 2019. “Dynamic Range Expansion Leads to Establishment of a New, Genetically Distinct Wolf Population in Central Europe.” Scientific Reports 9: 19003. 10.1038/s41598-019-55273-w. PMC6908625 31831858

[mec70639-bib-0075] Tallmon, D. A. , R. S. Waples , D. Gregovich , and M. K. Schwartz . 2012. “Detecting Population Recovery Using Gametic Disequilibrium‐Based Effective Population Size Estimates.” Conservation Genetics Resources 4, no. 4: 987–989. 10.1007/s12686-012-9689-3.

[mec70639-bib-0076] Tarroso, P. , G. Velo‐Antón , and S. B. Carvalho . 2015. “Phylin: An R Package for Phylogeographic Interpolation.” Molecular Ecology Resources 15: 349–357. 10.1111/1755-0998.12312. 25132358

[mec70639-bib-0077] Theissinger, K. , C. Fernandes , G. Formenti , et al. 2023. “How Genomics Can Help Biodiversity Conservation.” Trends in Genetics 39, no. 7: 545–559. 10.1016/J.TIG.2023.01.005. 36801111

[mec70639-bib-0079] Vilà, C. , A. K. Sundqvist , Ø. Flagstad , et al. 2003. “Rescue of a Severely Bottlenecked Wolf ( *Canis lupus* ) Population by a Single Immigrant.” Proceedings of the Royal Society B: Biological Sciences 270, no. 1510: 91–97. 10.1098/rspb.2002.218. PMC1691214 12590776

[mec70639-bib-0081] vonholdt, B. M. , D. R. Stahler , D. W. Smith , D. A. Earl , J. P. Pollinger , and R. K. Wayne . 2008. “The Genealogy and Genetic Viability of Reintroduced Yellowstone Grey Wolves.” Molecular Ecology 17, no. 1: 252–274. 10.1111/J.1365-294X.2007.03468.X. 17877715

[mec70639-bib-0082] Waples, R. S. 2006. “A Bias Correction for Estimates of Effective Population Size Based on Linkage Disequilibrium at Unlinked Gene Loci.” Conservation Genetics 7, no. 2: 167–184. 10.1007/s10592-005-9100-y.

[mec70639-bib-0083] Willi, Y. , T. N. Kristensen , C. M. Sgro , A. R. Weeks , M. Ørsted , and A. A. Hoffmann . 2022. “Conservation Genetics as a Management Tool: The Five Best‐Supported Paradigms to Assist the Management of Threatened Species.” Proceedings of the National Academy of Sciences of the United States of America 119, no. 1: e2105076119. 10.1073/PNAS.2105076119. PMC8740573 34930821

[mec70639-bib-0084] Wilson, G. A. , and B. Rannala . 2003. “Bayesian Inference of Recent Migration Rates Using Multilocus Genotypes.” Genetics 163, no. 3: 1177–1191. 10.1093/genetics/163.3.1177. PMC1462502 12663554

[mec70639-bib-0085] Wolf Alpine Group . 2024. “The Wolf Alpine Population in 2020–2024 Over 7 Countries.” Technical report for LIFE WolfAlps EU project LIFE18 NAT/IT/000972, Action C4.

