# Supplementary material for: From Founder Events to Genetic Recovery? Three Decades of Wolf Expansion Across the Italian Alps
Source: Mol Ecol. 2026 Nov 14;35(22):e70639. doi: 10.1111/mec.70639 (PMC13768827; doi:10.1111/mec.70639)
Supplement: Supplementary file 1 [file MEC-35-e70639-s001.docx]

**Supplementary S1. Methods and results of effective population size (N̂_e_) estimation for the Italian Alpine wolf population, 1998-2024.** Methods and results of N̂_e_ estimation using Linkage Disequilibrium and Sibship Frequency approaches, including sensitivity tests on generation length and starting year (Tables S1-S3) and estimates by cohort for the western and eastern Alps (Table S4).

From Founder Events to Genetic Recovery? Three Decades of Wolf Expansion Across the Italian Alps

Francesca Rolle^1*^; Kristine L. Pilgrim^2^; Elisa Avanzinelli^3^; Michael K. Schwartz^2^; Francesca Marucco^1^

^1^ Department of Life Sciences and Systems Biology, University of Torino, Torino, Italy

^2^ USDA National Genomics Center for Wildlife and Fish Conservation, Missoula, Montana, USA

^3^ Large Carnivore Center, Ente di Gestione delle Aree Protette delle Alpi Marittime, Valdieri, Italy

*Correspondence:

Francesca Rolle

Department of Life Sciences and Systems Biology, University of Torino, Via Accademia Albertina 13, 10123 Torino, Italy

[f.rolle@unito.it](mailto:f.rolle@unito.it)

1. Effective Population Size analysis methods and results

To account for temporal variability in the data and ensure robust estimates of the effective population size (N̂_e_), we first focused on the period 1998-2010, during which annual systematic genetic sampling was conducted. For this initial dataset, we estimated N̂_e_ using two different generation lengths: 3 years (Table S1) and 4 years (Table S2). This allowed us to evaluate how sensitive N̂_e_ estimates were to different generation intervals. To further reduce potential biases arising from the choice of the starting year, we implemented a third temporal grouping by shifting the starting year by one year (Table S3). This approach ensured that our N̂_e_ estimates were not strongly influenced by arbitrary choices in the temporal partitioning of the dataset. Following these sensitivity tests, we extended N̂_e_ calculations to the remaining years of data beyond 2010. Data were also stratified spatially based on the population structure results obtained in previous analyses (Table S4).

For each temporal pool, we applied three microsatellite-based methods to estimate N̂_e_: (i) Linkage Disequilibrium (LD) Method. Based on the method of Hill (1981) and Waples (2006), this approach estimates N̂_e_ from the non-random association of alleles at different loci. Calculations were performed using NeEstimator V2.1 (Do et al., 2014), under the assumption of random mating. Sibship Frequency (SF) method under (ii) random mating and (iii) non-random mating assumptions. Implemented in COLONY 2.0 (Jones & Wang, 2010), this method reconstructs pack relationships and estimates N̂_e_ from the proportion of full and half-sibling pairs.

Table S1. Estimates of N̂_e_ of the Italian Alpine wolf population, sampled as four cohorts of three years each between 1998 and 2010. SF rm: Sibship N̂_e_ assuming random mating; SF nrm: Sibship N̂_e_ assuming non random mating; LD: Linkage Disequilibrium N̂_e_.

| **Area** | **Cohort** | **SF rm**  (95% CI) | **SF nrm**  (95% CI) | **LD**  (95% CI) |
| --- | --- | --- | --- | --- |
| west Alps | 1998-2001 | **13** (7-30) | **18** (10-35) | **9.3** (4.7-15.3) |
| west Alps | 2001-2004 | **26** (16-44) | **27** (17-47) | **23.2** (16.2-34.1) |
| west Alps | 2004-2007 | **29** (18-50) | **31** (19-54) | **17.9** (13.6-23.3) |
| west Alps | 2007-2010 | **23** (13-42) | **29** (17-51) | **11.5** (8.2-15.8) |

Table S2. Estimates of N̂_e_ of the Italian Alpine wolf population, sampled as three cohorts of four years each between 1998 and 2009. SF rm: Sibship N̂_e_ assuming random mating; SF nrm: Sibship N̂_e_ assuming non random mating; LD: Linkage Disequilibrium N̂_e_.

| **Area** | **Cohort** | **SF rm** | **SF nrm** | **LD** |
| --- | --- | --- | --- | --- |
| west Alps | 1998-2002 | **13** (7-30) | **18** (10-35) | **10.2** (6.3-15.6) |
| west Alps | 2002-2006 | **29** (18-50) | **31** (19-53) | **19.4** (14.4-26.3) |
| west Alps | 2006-2009 | **21** (13-42) | **25** (16-46) | **12.7** (9.4-17.0) |

Table S3. Estimates of N̂e of the Italian Alpine wolf population, sampled as four cohorts of three years each between 1999 and 2010. SF rm: Sibship N̂_e_ assuming random mating; SF nrm: Sibship N̂_e_ assuming non random mating; LD: Linkage Disequilibrium N̂_e_.

| **Area** | **Cohort** | **SF rm** | **SF nrm** | **LD** |
| --- | --- | --- | --- | --- |
| west Alps | 1999-2002 | **17** (9-34) | **24** (14-44) | **9.3** (4.7-15.3) |
| west Alps | 2002-2005 | **29** (18-50) | **31** (19-53) | **19.4** (14.1-26.3) |
| west Alps | 2005-2008 | **24** (14-42) | **28** (18-48) | **16.2** (12.3-21.2) |
| west Alps | 2008-2009 | **20** (12-36) | **24** (14-44) | **12.5** (8.1-19.3) |

Table S4. Estimates of effective population size (N̂_e_) for the Italian Alpine wolf population, calculated from three-year cohorts spanning 1998–2024, with separate analyses for the western and eastern Alps.. SF rm: Sibship N̂_e_ assuming random mating; SF nrm: Sibship N̂_e_ assuming non random mating; LD rm: Linkage Disequilibrium N̂_e_ assuming random mating; LD m: Linkage Disequilibrium N̂_e_ assuming monogamy The number of packs observed in each time period is also reported.

| **Area** | **Cohort** | **SF rm**  (95% CI) | **SF nrm**  (95% CI) | **LD rm**  (95% CI) | **LD m**  (95% CI) | **N° packs** |
| --- | --- | --- | --- | --- | --- | --- |
| west Alps | 1998-2001 | **13** (7-30) | **18** (10-35) | **9.3** (4.7-15.3) | **15.4** (9.5-25.7) | **9** |
| west Alps | 2001-2004 | **26** (16-44) | **27** (17-47) | **23.2** (16.2-34.1) | **44.7** (30.3-69.3) | **12** |
| west Alps | 2004-2007 | **29** (18-50) | **31** (19-54) | **17.9** (13.6-23.3) | **39.2** (29.7-52) | **21** |
| west Alps | 2007-2010 | **23** (13-42) | **29** (17-51) | **11.5** (8.2-15.8) | **26.1** (19.7-34.6) | **16** |
| west Alps | 2014-2015 | **36** (23-59) | **35** (23-57) | **21.4** (16.4-28.0) | **45.8** (35.6-59.4) | **22** |
| west Alps | 2017-2018 | **64** (46-93) | **61** (42-90) | **54.1** (42.5-69.7) | **109.3** (86.4-140.2) | **38** |
| west Alps | 2020-2021 | **118** (91-153) | **111** (87-145) | **83.3** (67.6-103.4) | **165.9** (135.1-205.2) | **66** |
| west Alps | 2023-2024 | **161** (127-200) | **152** (123-193) | **122.2** (97.7-154.6) | **251.8** (201.2-319.2) | **/** |
| east Alps | 2014-2015 | **3** (2-∞) | **4** (2-20) | **3.1** (2.1-8.3) | **10.2** (4.2-19.2) | **/** |
| east Alps | 2017-2018 | **13** (7-30) | **13** (7-30) | **20.1** (12.3-35.4) | **41.9** (26.7-72.4) | **8** |
| east Alps | 2020-2021 | **30** (18-50) | **30** (19-53) | **24.6** (18.2-33.7) | **50.9** (38.2-69.0) | **20** |
| east Alps | 2023-2024 | **25** (15-46) | **24** (15-43) | **42.7** (25.7-86.2) | **86.7** (53.0-172.9) | **/** |

References

Do, C., Waples, R. S., Peel, D., Macbeth, G. M., Tillett, B. J. & Ovenden, J. R. (2014). NeEstimator V2: re-implementation of software for the estimation of contemporary effective population size (N_e_) from genetic data. Molecular Ecology Resources. 14, 209-214. https://doi.org/10.1111/1755-0998.12157

Hill WG. (1981). Estimation of effective population size from data on linkage disequilibrium. *Genetical Research,* 38(3), 209-216. <https://doi.org/10.1017/S0016672300020553>

Jones, O.R., Wang, J. (2010), COLONY: a program for parentage and sibship inference from multilocus genotype data. *Molecular Ecology Resources*, 10, 551-555. https://doi.org/10.1111/j.1755-0998.2009.02787.x

Waples, R. S. (2006). A bias correction for estimates of effective population size based on linkage disequilibrium at unlinked gene loci. Conservation Genetics, 7(2), 167-184. https://doi.org/10.1007/s10592-005-9100-y
